# Effect of the COVID-19 pandemic on health service utilization across regions of Ethiopia: An interrupted time series analysis of health information system data from 2019–2020

**DOI:** 10.1371/journal.pgph.0000843

**Published:** 2022-09-12

**Authors:** Anagaw Derseh Mebratie, Adiam Nega, Anna Gage, Damen Haile Mariam, Munir Kassa Eshetu, Catherine Arsenault

**Affiliations:** 1 School of Public Health, Addis Ababa University, Addis Ababa, Ethiopia; 2 Department of Global Health and Population, Harvard T.H. Chan School of Public Health, Boston, MA, United States of America; 3 Minister’s Office, Ministry of Health of Ethiopia, Addis Ababa, Ethiopia; Institute of Public Health Bengaluru, INDIA

## Abstract

The spread of COVID-19 and associated deaths have remained low in Ethiopia. However, the pandemic could pose a public health crisis indirectly through disruptions in essential health services. The aim of this study was to examine disruptions in health service utilization during the first nine months of the COVID-19 pandemic across 10 regions in Ethiopia. We analyzed utilization of 21 different health services across all of Ethiopia (except the Tigray region) for the period of January 2019 to December 2020. Data were extracted from the Ethiopian district health information system (DHIS2). Monthly visits in 2020 were graphed relative to the same months in 2019. Interrupted time series analysis was used to estimate the effect of the pandemic on service utilization in each region. We found that disruptions in health services were generally higher in urban regions which were most affected by COVID. Outpatient visits declined by 52%, 54%, and 58%, specifically in Dire Dawa, Addis Ababa and Harari, the three urban regions. Similarly, there was a 47% reduction in inpatient admissions in Addis Ababa. In agrarian regions, the pandemic caused an 11% to 17% reduction in outpatient visits and a 10% to 27% decline in inpatient admissions. Visits for children with diarrhea, pneumonia and malnutrition also declined substantially while maternal health services were less affected. Our study indicates that disruptions in health services were more pronounced in areas that were relatively harder hit by the pandemic. Our results show that the Ethiopian health system has a limited capacity to absorb shocks. During future waves of COVID or future pandemics, the Ethiopian health system must be better prepared to maintain essential services and mitigate the indirect impact of the pandemic on public health, particularly in urban areas.

## Introduction

In a time of crisis, health systems need to both respond to the outbreak and maintain the provision of other essential health services. The spread of COVID-19 across African countries in 2020 and its direct health impacts were not as severe as many had feared. In Ethiopia, only 1,054 COVID cases per million were reported and only 16 people per million died from the condition in 2020 [1]. However, there is a growing concern that by disrupting essential health services, the pandemic could have important consequences on population health [2, 3]. A reduction in utilization of essential health services could have long-term effects by increasing chronic illnesses, disability or preventable mortality [4]. In addition, certain population groups risk being systematically more affected resulting in widening health inequalities.

The Ethiopian health system, which has historically been under-funded and under-resourced, may be particularly vulnerable during the COVID-19 pandemic. The pandemic could result in worse health outcomes due to reduced service provision, declines in quality of care, or poorer healthcare seeking behavior. Other health systems have reported lower volumes of non-COVID-19 patients and critical declines in preventive services such as immunizations [5]. Before the pandemic, access to quality care was already a challenge in Sub-Saharan Africa. Health providers performed less than half of the most basic elements of recommended care and facilities often lacked necessary equipment and supplies [6].

Ethiopia was able to prevent widespread transmission of COVID-19. After the first case was reported in mid-March 2020, the government declared a five-month national state of emergency starting April 8th, 2020. Restrictions on large gatherings and movement were imposed, most civil servants were asked to work from home, and the closure of schools was ordered. The government also suspended flights to 120 countries and granted a pardon to 20,402 prisoners to reduce overcrowding in prisons [7]. The country also put in place a 14-day mandatory quarantine and COVID test for inbound travelers. Mass media communication campaigns were developed to promote hand washing, mask wearing, and social distancing. When COVID continued to spread in Addis Ababa and other urban areas, the government imposed mandatory facemasks in public places and reduced the number of passengers allowed in public transports. Night clubs, concerts, cinema houses, churches and mosques were also closed, and sport events were cancelled in the capital. In addition, the Ethiopian Federal Ministry of Health and its technical arm, the Ethiopian Public Health Institution, established a surveillance mechanism as per WHO recommendations to regularly check the status of COVID-19 in the population and disseminate health-related information [8].

Some of the COVID-19 prevention measures taken in Ethiopia could have had adverse impacts on the utilization of essential health services. Prior to the pandemic, health care utilization and health outcomes were relatively poor in Ethiopia compared to other countries. For example, only 48% of women gave birth in health facilities, and the newborn mortality rate was 30 per 1,000 live births [9]. Health intervention coverage and health outcomes also varied widely across regions in the country, with stark disparities between the capital and other areas. The conversion of selected health facilities into COVID-19 treatment centers and the redeployment of health workers to COVID-19 treatment centers could have also reduced access to and provision of essential health services [10]. Moreover, due to limited understanding about the disease and limited preparedness of health facilities, the community could fear being exposed to the virus while visiting health facilities and avoid or delay seeking care. Moreover, the pandemic could severely affect some segments of the population and increase health inequalities.

Although increasing attention is being paid to assess how health systems should respond to the pandemic, less is known about the effect of the pandemic on non-COVID health services. Previous analyses have quantified the effect of COVID on health service utilization in Ethiopia at the national level, but there is limited evidence on whether these effects have differed across sub-national areas or across different types of services [11]. Our study assesses the trends in a range of essential health services during the first nine months of the COVID-19 pandemic across regions in Ethiopia and identifies the worst and least affected regions.

## Materials and methods

### Study setting

Ethiopia is the second most populous country in Africa with more than 114 million estimated population size in 2020 [12, 13]. Over the last two decades, in order to improve health outcomes, the country has given due attention to improve the health system and made considerable investments to expand access to health services. According to a report by the Ministry of Health (2019), Ethiopia has 16,660 health posts, 3,622 health centers, and 266 public hospitals. Moreover, there are 7,576 clinics and 62 hospitals owned by the private sector [14]. Consequently, primary health service coverage, as measured by access to a health facility within a two-hour walk, has incresed from 51% in 2001 to 98% in 2016 [15].

Despite the recent progress made in expanding access to essential health services, there are notable variations in spatial distribution of healthcare infrastructure across regions in Ethiopia. People in rural and pastoralist areas generally have limited access to services compared to urban residents. According to the 2019 Health and Health Indicators report by the Federal Ministry of Health, the average number of people served per one hospital in Addis Ababa is 164,998 and 48,583 in Harari. On the other hand, one hospital in Afar, Oromia and Somali serve on average 331,468, 302,757 and 581,126 people, respectively [16, 17]. Furthermore, private clinics are largely concentrated in urban areas. Similarly, the number of nurses per 100,000 population is 162, 155 and 123 in Gambella, Harari and Addis Ababa compared to only 4.8, 5.1 and 5.5 in Oromia, Somali and Amhara. The number of medical doctors per 100,000 population ranges from 2.6 in Oromia to 24.1 in Harari. The corresponding figure for Addis Ababa is 13.9 [16, 18].

Service utilization also varies across regions. In 2019, outpatient attendance per capita per year in urban areas ranged from 1.15 to 1.74. In contrast, outpatient attendance was lowest in pastoralist regions namely Afar (0.36) and Somali (0.21) [16, 19].

### Study design and timelines

We conducted an interrupted time series study using health management information system data to address the research objective. Two years of essential service data for the period of Tirr 1,2011to Tahsas 30, 2013 (Equivalent to January 9, 2019 to January 8, 2021) was used for the analysis.

### Data source

The data were extracted from the Ethiopian District Health Information System 2 (DHIS2) and provided by the Ethiopian Ministry of Health. The study covers 8 regions (Afar, Amhara, Oromia, Somali, SNNP, Gambella, Harari and Benishangul Gumz) and 2 city administrations (Addis Ababa and Dire Dawa) in Ethiopia. The study excludes Tigray region because services utilization data were missing for the last three months of the study period (between October and December, 2020) due to the ongoing conflict in the region.

The dataset combined facility-level and woreda (district)-level data: facility-level information was available for tertiary and secondary hospitals and private facilities and for all facilities in the capital, Addis Ababa. Data from other public facilities (primary hospitals, health centers and health posts) were aggregated and reported by woreda health offices in all regions except in the capital, Addis Ababa.

### Outcome measures

We assessed utilization for various types of health services including maternal health services, child vaccinations, inpatient and outpatient care and family planning service (See **Table D in S1 Appendix**). We analyzed the absolute number of monthly visits or services provided rather than service coverage indicators (i.e., proportion of target population who received a specific service) as the later can be unreliable because they depend on estimated target population sizes as denominators.

### Data cleaning

Using DHIS2 data during the pandemic could face challenges with data completeness and reporting delays. To ensure that our dataset included a stable number of facilities reporting each month, we included (for each indicator) only health facilities (or woreda health offices) that reported non-missing values for at least 15 months out of 24. This threshold was chosen because it best balanced the need for stable reporting without excluding too much data.

Second, positive outliers were identified and replaced to missing. Outliers are utilization counts greater than 3.5 standard deviation from the facility mean over 24 months. Negative outliers (values lower than 3.5 standard deviations from the mean), were not removed as utilization may have reasonably decreased during the pandemic to low levels. Finally, datasets were compared before and after the two steps mentioned above to make sure that the data cleaning did not bias the dataset. Then the sum of each indicator in the raw vs. cleaned dataset was compared to ensure that cleaning procedures did not exclude too much data. Further details on data cleaning and data validation procedures were published previously [11].

### Data analysis

For the analysis, service volumes were aggregated at the regional level on a monthly basis. We first presented results descriptively using trend graphs showing the volume of each service during COVID compared to the same month pre-COVID, by region. Second, interrupted time series analysis (ITSA) was used to estimate the level and slope change during the first nine months of the pandemic. April 2020 was used as the interruption period. In addition, to the regional-level estimates, we also repeated the analysis by aggregating services by region type. We categorized the regions and city administrations into three groups. These included urban regions (Addis Ababa, Dire Dawa, and Harari), agrarian regions (Amhara, Oromia and SNNP) and pastoralist regions (Benishangul-Gumuz, Gambella, Afar and Somali). These region types are widely used in Ethiopia for policy making and research. To compare coefficients from linear models across regions and health services, we created a relative level change estimate by dividing the estimate for the level change by the average service volume in the pre-COVID period (from January 2019 to March 2020).

The interrupted time series analysis predicts two types of coefficients indicating the effect of the pandemic on utilization of care for each type of health service. The first one is a level change in volume of visits showing the monthly average service visits immediately after the declaration of the pandemic in April 2020 minus the monthly average visits during pre-COVID-19. The second estimate is a slope change in healthcare use that indicates the magnitude of change in the volume of monthly average service utilization during COVID-19 period until the end of 2020. In the ITSA, estimates were considered to be statistically significant if the p-value ≤ 0.05. All analyses were conducted using Stata version 16.0 (StataCorp, College Station, Texas 77845 USA).

We also conducted two sensitivity analysis to confirm consistency of the estimates. First, we used a prais regression model where the errors are assumed to follow a first-order autoregressive process to check whether results were consistent. We also repeated the analysis by including dummies for quarters of the year in order to account for seasonality (potential changes in demand for services at different times of the year).

### Ethics

This study was conducted using secondary data accessed from the Ethiopian DHIS-2. The Federal Ministry of Health approved the research project. In addition, ethical approval and clearance was obtained from the Institutional Review Board of the College of Health Science of Addis Ababa University (IRB-CHS). We received de-identified and anonymized data from DHIS2. Data were not copied from or removed from the data repository prepared by the Harvard T.H. Chan School of Public Health without prior permission from the local country PI and they were not shared with anyone except the members of the research team at Harvard University and Addis Ababa University.

## Results

We analyzed a total of 21 types of health services included in the Ethiopian DHIS2. **Table A in S1 Appendix** shows the number of units (facilities, Woredas or primary health center units) included in the study across regions. The majority of facilities are found in Oromia, the largest region in Ethiopia, followed by the Southern Nations, Nationalities and People’s (SNNP) region.

**Fig 1:**
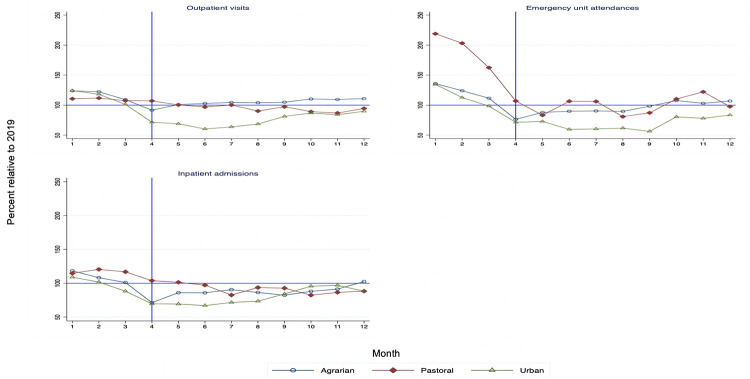
Overall service utlization in 2020 compared to 2019 by region type.

**Fig 2:**
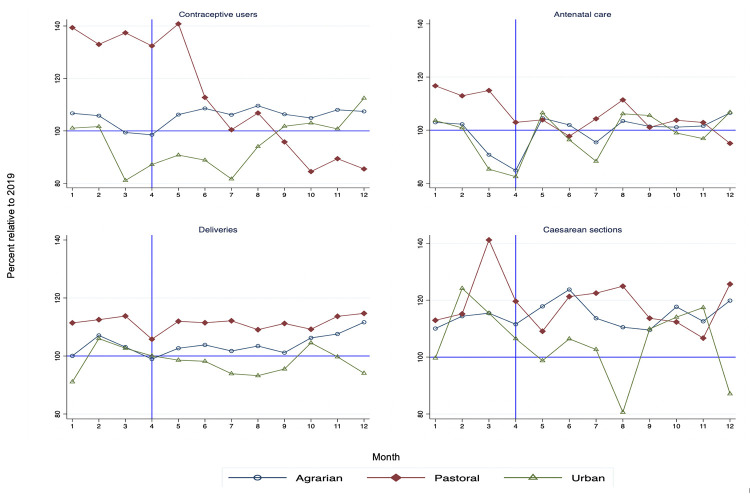
Reproductive and maternal health services in 2020 compared to 2019 by region type.

**Fig 3:**
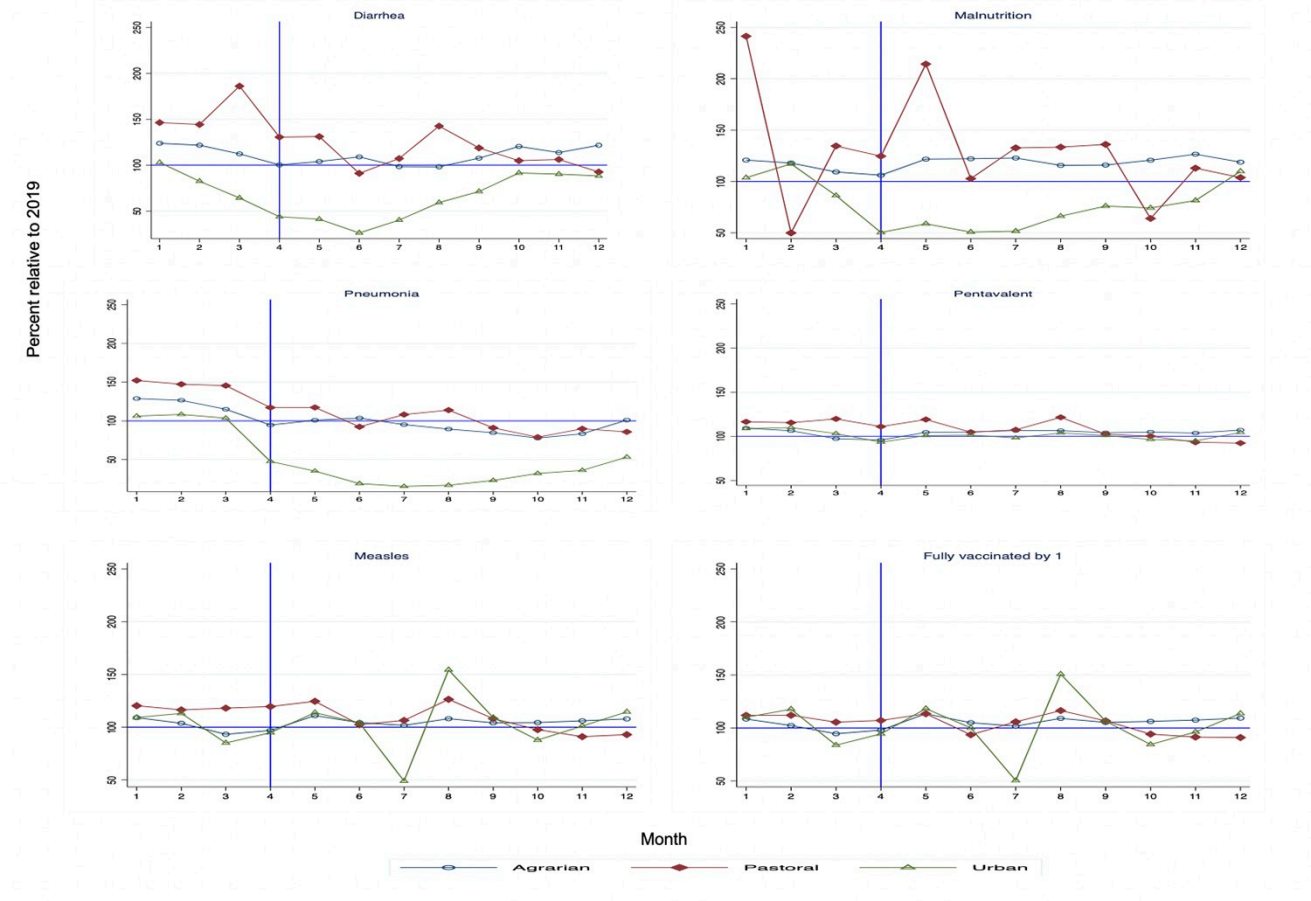
Child health services and vaccinations in 2020 compared to 2019 by region type.

Relative service utilization (service volume in 2020 compared to the same month in 2019) across the three region types are shown in **Figs 1–3**. Estimates for the immediate level and the slope change during the pandemic with confidence intervals and p-values are reported in **Tables 1–3**. In these tables, the level change estimates indicate the effect of the pandemic immediately following the declaration of the pandemic while the slope change refers to the monthly change in visits during the pandemic period until the end of 2020. For improved interpretability, the relative level change as compared to the average in the pre-COVID-19 period is also reported.

**Table 1:** Effect of the COVID-19 pandemic on overall service utilization in each region (ITSA—Regression with Newey-West standard errors).

Region	Outpatient visits			Emergency room visits			Inpatient admissions		
	Level change (95% CI)	Relative level change	Slope change (95% CI)	Level change (95% CI)	Relative level change	Slope change (95% CI)	Level change (95% CI)	Relative level change	Slope change (95% CI)
Addis Ababa	-317,757** (-417,938, -217,576)	-54%	22,732** (11,793, 33,671)	-14,156** (-18,272, -10,040)	-47%	662** (131, 1,194)	-4,532** (-6,436, -2,627)	-34%	497** (224, 769)
Afar	-15,157** (-24,084, -6,229)	-25%	783 ( -380, 1,946)	-538 (-1,129, 53)	-34%	83 (-19, 185)	-287** (-459, -115)	-27%	11(-6, 27)
Amhara	-654,667** (-918,256, -391,077)	-27%	50,757** (8,721, 92,793)	-26,887** (-32,107, -21,667)	-43%	1,979** (818, 3,141)	-7,702** (-1,0421, -4,985)	-35%	797** (454, 1,140)
Ben-Gum	-10,886 (-32,563, 10,791)	-14%	-1,324 (-3,999, 1,351)	-1,278** (-1,859, -699)	-62%	21 (-71- 111)	-448** (-729, -167)	-33%	16 (-25, 57)
Dire Dawa	-32,461** (-4062, -24294)	-52%	1,397** (541, 2,254)	-4,655** (-6,987, -2,324)	-98%	33 (-221, 288)	-529** (-956, -102)	-34%	83** (24, 142)
Gambella	-1,889 (-4,750, 972)	-13%	-236 (-688, 215)	-2,020** (-3,104, -937)	-64%	-226** (-409, -43)	-256 (-608, 96)	-29%	-3 (-58, 53)
Harari	-15,104** (-20,152, -10,057)	-58%	1,639** (767, 2,511)	-951** (-1,550, -353)	-41%	66 (-15, 146)	-397** (-737, -58)	-23%	65** (16, 114)
Oromia	-525,620** (-733,243, -317,998)	-24%	75,279** (48,332, 102,227)	-28,411** (-37,838, -18,985)	-43%	3,567** (2,218, 4,915)	-13,753** (-18,241, -9,266)	-39%	1,053** (562, 1,544)
SNNP	-163,068** (-250,758, -75,378)	-10%	33,659** (19,638, 47,681)	-17,880** (-23,070, -12,691)	-33%	1,324** (554, 2,095)	-6,467** (-8,124, -4809)	-37%	574** (382, 765)
Somali	-9,617 (-20,258, 1,025)	-8%	-194 (-1,867, 1,478)	-1,120** (-2,033, -208)	-57%	147** (18, 276)	-1,135** (-1,666, -603)	-23%	-50 (-107, 7)

Level change shows the immediate effect of the pandemic in April 2020 and slope change is the monthly change during COVID-19 until the end of 2020. Relative level change is the immediate level change in April 2020 compared to the average during the pre-COVID period (January 2019 to March 2020). Asterisks ** indicate statistical significance (p ≤0.05). SNNP is Southern Nations, Nationalities and People’s region. Ben-Gum is Benishangul-Gumuz region.

**Table 2:** Effect of the COVID-19 pandemic on reproductive and maternal health services by region (ITSA—Regression with Newey-West standard errors).

Region	Contraceptive users			Antenatal care			Deliveries			Caesarean sections		
	Level change (95% CI)	Relative level change	Slope change (95% CI)	Level change (95% CI)	Relative level change	Slope change (95% CI)	Level change (95% CI)	Relative level change	Slope change (95% CI)	Level change (95% CI)	Relative level change	Slope change (95% CI)
Addis Ababa	-5,179 (-8,353, -2,006)	-20%	1,114** (588, 1,640)	-1,853** (-3,276, -427)	-14%	319 (-38, 676)	9 (-82, 100)	0%	-79 (-412, 254)	-392 (-929,145)	-12%	-2 (-149, 145)
Afar	-737 (-1548, 73)	-8%	196** (99, 293)	-439** (-810, -68)	-10%	57 (-17, 132)	-107 (-262, 47)	-8%	29** (6, 52)	8 (-8, 23)	16%	-2** (-5, 0)
Amhara	-8,665 (-42,704, 25,374)	-3%	3,987(-4293, 12,266)	-9,047** (-17,856, -238)	-16%	1,624 (-153, 3,401)	-1,732 (-4,126, 662)	-5%	-95 (-375, 185)	-174 (-407, 59)	-8%	-4 (-44, 36)
Ben-Gum	-846 (-1,967, 274)	-9%	-64(-299, 172)	-324 (-727, 78)	-10%	-15 (-98, 67)	-48 (-208, 112)	-3%	8 (-13, 30)	18 (-5, 42)	17%	-3 (-7, 1)
Dire Dawa	-375 (-803, 52)	-10%	116** (68, 163)	-126 (-354, 102)	-9%	50** (18, 81)	32 (-125, 188)	4%	36** (12, 60)	11 (-25, 47)	7%	7 (-3, 16)
Gambella	-350 (-798, 98)	-24%	119** (75, 162)	-207** (-338, -76)	-25%	42** (20, 64)	-5 (-66, 56)	-1%	5 (-5, 16)	-7 (-17, 4)	-25%	-1 (-3, 1)
Harari	-628** (-1,059, -197)	-24%	147** (68, 226)	-240** (-453, -27)	-27%	55 ** (24, 85)	82 (-95, 259)	10%	2 (-24, 28)	31(-7, 69)	17%	-2 (7, 2)
Oromia	-26,327 (-58,287, 5,633)	-6%	8,186** (1,295, 15,078)	-17,669** (-30,111, -5,227)	-14%	3,164** (536, 5791)	-4,370** (-7,768, -972)	-7%	1,569** (1,118, 2,021)	19 (-186, 224)	1%	49** (22, 76)
SNNP	3,162(-28,183, 34,507)	1%	298(-5,439, -5439)	-4,140 (-8,405, 124)	-7%	989** (119, 1859)	-1,493 (-3,529, 543)	-4%	351** (136, 566)	24 (-149, 197)	1%	-9 (-43, 25)
Somali	-1,718 (-7,841, 4,404)	-13%	-2,187** (-3,234, -1,139)	-1,141 (-2,370, 87)	-7%	-257 (-475, -38)	-172 (-476, 133)	-3%	24 (-38, 87)	-14 (-49, 20)	-10%	4 (-2, 9)

Level change shows the immediate effect of the pandemic in April 2020 and slope change is the monthly change during COVID-19 until the end of 2020. Relative level change is the immediate level change in April 2020 compared to the average during the pre-COVID period (January 2019 to March 2020). Asterisks ** indicate statistical significance (p ≤0.05). SNNP is Southern Nations, Nationalities and People’s region. Ben-Gum is Benishangul-Gumuz region

**Table 3:** Effect of the COVID-19 pandemic on child health services and vaccinations by region (ITSA—Regression with Newey-West standard errors).

Region	Diarrhea			Malnutrition			Pneumonia			Pentavalent			Measles			Fully vaccinated by 1		
	Level change (95% CI)	Relative level change	Slope change (95% CI)	Level change (95% CI)	Relative level change	Slope change (95% CI)	Level change (95% CI)	Relative level change	Slope change (95% CI)	Level change (95% CI)	Relative level change	Slope change (95% CI)	Level change (95% CI)	Relative level change	Slope change (95% CI)	Level change (95% CI)	Relative level change	Slope change (95% CI)
Addis Ababa	-715** (-1,290, -139)	-38%	132** (38, 226)	-32,736** (-39,228, -26,244)	-62%	2,466** (1,464, 3,468)	-3,881** (-5,464, -2,297)	-110%	45 (-123, 213)	-1,084 (-2,644, 475)	-10%	66 (-177, 308)	-614 (-3,791, 2,564)	-6%	124 (-230, 478)	-594 (-2,620, 1,431)	-6%	77 (-235, 388)
Afar	57 (-78, 192)	7%	11 (-25, 46)	-2,418 (-10,150, 5,314)	-22%	364 (-10,000, 1,728)	-814** (-1,350, -277)	-42%	5 (-64, 75)	-389** (-673, -104)	-12%	-2 (-50, 46)	-239 (-580, 102)	-8%	-59** (-110, -8)	68 (-163, 300)	3%	1 (-35, 36)
Amhara	-1,034** (-1,984, -84)	-17%	170** (51, 290)	-119,584 (-262,268, 23,100)	-12%	35,340** (11,176, 59,505)	-21,051** (-28,732, -13,370)	-64%	792 (-129, 1,713)	-1,117 (-7,045, 4,811)	-2%	120 (-835, 1,075)	-59 (-9,033, 8,915)	0%	10 (-1,406, 1,427)	239 (-7,967, 8,446)	0%	112 (-1,178, 1,402)
Ben Gum	-156 (-371, 60)	-22%	-36** (-71, -1)	9759 (-34,948, 54,466)	17%	-2,802 (-10,373, 4,770)	-1,543 ** (-2,461, -626)	-58%	-91 (-193, 12)	122 (-304, 548)	5%	-65(-148, 19)	112 (-436, 660)	5%	-40 (-125, 45)	73 (-377, 523)	3%	-48 (-116, 21)
Dire Dawa	-124** (-214, -34)	-52%	14** (3, 24)	-1,766** (-3,082, -450)	-43%	552** (193, 910)	-145** (-220, -70)	-52%	12 (-8, 32)	-305** (-413, -198)	-30%	38** (22, 53)	-166** (-306, -26)	-18%	5 (-18, 27)	-147** (-241, -54)	-17%	-1 (-21, 20)
Gambella	-55** (-107, -2)	-38%	9** (1, 16)	-4,033 (-16,901, 8,835)	-49%	-393 (-1,746, 959)	-482** (-780, -184)	-77%	27 (-32, 87)	-97 (-219, 23)	-10%	37** (13, 62)	70 (-105, 246)	9%	36** (2, 69)	64 (-76, 204)	10%	5 (-22, 31)
Harari	-6 (-68, 55)	-4%	7 (-9, 22)	-6,836** (-10,507, -3,165)	-81%	1,633** (844, 2,422)	-212** (-362, -61)	-82%	40** (13, 66)	-121 (-252, 10)	-18%	31** (12, 49)	-106 (-289, 78)	-16%	43** (14, 72)	-139 (-300, 21)	-24%	40** (15, 64)
Oromia	-2,971** (-5,879, -63)	-13%	401(-157, 959)	-265,179 (-552,967, 22,609)	-10%	64,892** (24,851, 104,933)	-35,571** (-51,885, -19,256)	-44%	1,634 (-65, 3,333)	-6,647 (-15,805, 2,511)	-6%	1,782** (439, 3,125)	-4,265 (-18,045, 9,516)	-4%	1,483 (-565, 3,530)	-3,253 (-14,191, 7,687)	-4%	1,577 (-229, 3,384)
SNNP	-1,620** (-2,324, -916)	-24%	67 (-51, 186)	-57,424** (-107,298, -7,550)	-5%	26,439** (16,684, 36,193)	-15,440** (-22,725, -8,155)	-44%	806** (31, 1,580)	149 (-4,751, 5,050)	0%	170 (-611, 950)	1,396 (-4,420, 7,211)	3%	163 (-770, 1,096)	803 (-4,310, 5,916)	2%	210 (-615, 1,037)
Somali	-769 (-2,294, 758)	-15%	-281** (-526, -37)	-3,540 (-9,344, 2,263)	-11%	953 (-186, 2,092)	-1,501** (-2,443, -559)	-19%	-282** (-436, -128)	-787 (-1,834, 260)	-5%	-420** (-569, -272)	-557 (-1,526, 412)	-4%	-450** (-601, -300)	-935** (-1,625, -246)	-9%	-216** (-325, -108)

Level change shows the immediate effect of the pandemic in April 2020 and slope change is the monthly change during COVID-19 until the end of 2020. Relative level change is the immediate level change in April 2020 compared to the average during the pre-COVID period (January 2019 to March 2020). Asterisks ** indicate statistical significance (p ≤0.05). SNNP is Southern Nations, Nationalities and People’s region. Ben-Gum is Benishangul-Gumuz region

### Overall utilization of health services

**Fig 1** shows the trends in outpatient visits, inpatient admissions and emergency room visits in 2020 compared to 2019. Between April and June 2020, outpatient visits were substantially lower in urban regions (Addis Ababa, Dire Dawa and Harari). In June 2020, the volume of outpatient visits in these urban regions was only 60% on average of that in June 2019. In pastoral regions (Afar, Gambella, Benishangul-Gumuz and Somali), outpatient care continued to fall until November 2020. In agrarian regions (that is Amhara, Oromia and SNNP), outpatient visits did not appear to be affected. Similarly, there were declining trends in emergency room visits and inpatient admissions during the first few months of the pandemic. Overall, reductions in these three services were relatively higher in urban areas.

Results from interrupted time series regressions show that COVID-19 disrupted outpatient visits use immediately following the declaration of the state of emergency in Ethiopia. Reductions in outpatient visits were significant at 5% level of significance in all regions except in pastoralist areas. Immediate effects ranged from a 10% reduction in SNNP to a 58% decline in Harari region (**Table 1**). In the capital, Addis Ababa, outpatient visits declined by 54% in April 2020. The immediate effect of the pandemic was stronger in urban regions compared to agrarian regions. ITSA indicates that changes in service trends during COVID were significant and positive in most regions indicating a trend toward resumption in service use.

The volume of emergency unit attendances and inpatient admissions were also disrupted in April 2020 (**Table 1**). Declines were statistically significant in all regions expect in Afar and Gambella. In Dire Dawa, emergency unit attendances declined by 98% compared to the pre-COVID average. Slope changes during the pandemic were significant and positive in most regions except in Gambella where emergency unit attendances continued to decline.

### Reproductive and maternal services

The study also assessed reproductive and maternal health services during COVID-19 in Ethiopia. However, compared to the total outpatient visits and inpatient admissions, the disruptions in these services were smaller and rebounded to pre-COVID levels within a short period of time. For instance, **Fig 2** and **Table 2** show that institutional deliveries were not affected during the first nine months of the pandemic (except in Oromia where there was a brief 7% decline). In contrast, there was an overall positive trend in facility deliveries in agrarian and pastoralist areas.

A few regions faced reductions in other reproductive and maternal services. For instance, the number of contraceptive users significantly declined in Harari at the beginning of the pandemic. Similarly, visits for STIs declined in five out of the ten regions but returned to the pre-COVID levels a few months later (**Table B in S1 Appendix**). Caesarean sections were only moderately affected in Afar where they declined by 2.3 caesareans per month (**Table 2**).

### Child healthcare services

The study also looked at visits for child diarrhea, malnutrition, and pneumonia. **Fig 3** and regression analyses (**Table 3**) show that these services were affected in several regions. In particular, the number of children under 5 years who were treated for pneumonia declined substantially in all regions. For instance, in Addis Ababa, the relative level reduction in pneumonia visits compared to the pre-COVID average was 110% (**Table 3**). The slope change for pneumonia and diarrhea was generally positive except in Benishangul-Gumuz and the Ethiopian Somali regions where these services continued to decline.

### Child vaccinations

The study also investigated the effect of the pandemic on various child vaccines and on the number of children fully vaccinated by one year of age. Unlike curative services for sick children, child vaccinations were less affected by the pandemic (**Fig 3**, **Table 3**, and **Tables A-C in S1 Appendix**). Afar, Dire Dawa and Harari had declines in several vaccines. There was also a negative trend in all vaccines in Somali. The number of children fully vaccinated by age 1 declined substantially in Dire Dawa and Somali (**Table 3**).

### Other services

The study also examined the provision of antiretrovirals (ART) and the number of road traffic injuries treated in health facilities. The pandemic did not lead to a significant reduction in the number of adults and children receiving ART except in Gambella where ART declined by 16% in April 2020 (**Table C in S1 Appendix**). On the other hand, there were immediate and significant reductions in the number of people treated for road traffic accidents in half of Ethiopian regions. Relative level reductions ranged from 18% in Oromia to 92% in Harari (**Table C in S1 Appendix**).

Results from analyses using prais-winsten regressions and those that accounted for seasonality were consistent with results from the main analysis.

## Discussion

In this study, we used DHIS2 data from all of Ethiopia (with the exception of the Tigray region) to assess the effect of the pandemic on health care utilization across sub-national regions. Prior analyses found only small reductions in health service use at the national level in Ethiopia. In this paper, we found substantial heterogeneity in the impact of the pandemic on health services across regions. The three urban regions in Ethiopia (Addis Ababa, Dire Dawa and Harari) were the most affected. For example, the immediate decline in outpatient visits ranged from 52% to 58% in urban areas while declines ranged from 10% to 27% in agrarian regions. Across the study regions, it seems that the pandemic immediately disrupted utilization of services in urban and agrarian regions. On the other hand, the effect of the pandemic persisted overtime during the COVID period in some pastoralist regions (Afar, Benishangul-Gumuz, Gambella and Somali) which had statistically significant negative trends in several services. Our analysis reflects the importance of studying subnational differences in the impact of the pandemic on health services.

Variations in the spread of COVID and in the preventive measures taken across regions could explain the regional heterogeneities in service disruptions. For instance, by August 16, 2020, about 62% of national COVID cases were reported in Addis Ababa city. On the other hand, the share of confirmed cases in pastoralist regions including Afar, Gambella and Benishangul Gumz was less than 5% (**Table A in S1 Appendix**) [20]. Accordingly, COVID-19 preventive measures such as social distancing and restriction on movements were mainly enforced in urban areas. In addition, there was a limited awareness of the coronavirus and limited adoption of preventive measures in rural and remote areas of Ethiopia [21, 22]. Therefore, those who lived far away from urban areas seem to have continued their normal activities and to have visited health facilities without being concerned about the risk of contracting COVID. In addition, unlike in urban areas, the presence of community-based health services in rural Ethiopia could also contribute to maintaining service use during the pandemic. For instance, in different agrarian regions, health extension workers and volunteers conduct home visits to promote routine health services, check on pregnant women and provide immunization services [20, 23].

Important declines in service use in urban areas could also be partly attributed to economic factors. The pandemic led to worst disruptions in income-generating activities in urban areas. Agricultural activities were less affected during COVID-19. The pandemic mostly affected the service, manufacturing and construction sectors. Many people in urban areas lost their jobs, and their incomes were considerably reduced. It is estimated that the pandemic led to a 14% job loss in the service sector alone and to a 3.5 percentage-point increase in the poverty headcount ratio at the national level. Consequently, consumption expenditures including health spending among households (especially those in informal urban sectors) were severely disrupted [24–26]. The increase in the cost of public transports (due to the mandated reduction in passenger volumes) also played a role in reducing health care use in urban areas by increasing direct non-medical costs [27, 28]. Furthermore, although government health expenditure as a share of total expenditures reached 10% for the first time in 2020, most of these resources were directed to the COVID-19 response. A reduction in the availability of health services that were offered free of charge in public facilities has been reported [29].

We also found that different services were differently affected. Outpatient visits, emergency attendance and inpatient admissions were disrupted in almost all regions of the country. Visits for STIs also declined substantially in half the regions. Curative services for sick children (ORS for diarrhea, child pneumonia visits and malnutrition visits) also declined substantially. In the case of diarrhea and pneumonia, declines may partially be explained by reduced disease incidence from social distancing, mask wearing and better handwashing.

The magnitude of disruptions in maternal health services and child vaccination were relatively lower and there were fewer regions reporting significant declines in these services. For instance, only two out of the ten study regions had significant declines in postnatal care visits. Facility delivery and caesarian sections were not disrupted (except in Oromia were facility deliveries declined by 7%). It is well known that facility delivery and caesarian sections are key services to reduce maternal complications and deaths and maintaining these services is crucial [30]. Similarly, child vaccinations were generally maintained except in Dire Dawa and Harari regions.

The relatively limited effects of COVID-19 on maternal health and child vaccinations services, could be due to previous health programs and better awareness of the importance of MCH services. Since 2003, the country has implemented the health extension program and health development army which focused on health education, outreach, and improving demand for maternal and child health services. These programs contributed to reductions in maternal and child mortality rates by increasing healthcare seeking behavior for maternal and child services especially in rural Ethiopia [31, 32]. After the incidence of COVID in Ethiopia, governmental and non-governmental organizations also disseminated information using mass media to promote the use of maternal and child health services by following the COVID-19 preventive measures [33].

The pandemic did not seem to affect the number of patients who were on ART in any of the study regions. However, there had been significant reduction in the volume of visits for road traffic injuries especially in urban and agrarian areas. Following the spread of the COVID-19 diseases, the government of Ethiopia announced travel restrictions especially in urban areas. This led to considerable reduction in daily vehicle movements and thereby the incidence of traffic injuries [34].

In addition to the immediate effect of COVID at the beginning of April 2020, the study also assessed the trends in services until the end of 2020.The change in slopes were positive for various indicators including outpatient, emergency attendance and inpatient admission. This implies a trend toward recovery in health services and that the disruptions caused by COVID-19 and lock down measures were not severe and long lasting. This is consistent with results from a previous study conducted in selected Ethiopian districts on essential health and nutrition services. By the end of 2020, the study showed that utilization of services had started to recover to pre-pandemic level [10]. A periodic facility assessment conducted by UNICEF (2020) also reported that the trends in service utilization had recovered after an initial decrease in Oromia, Amhara, SNNP and Somalia regions [35].

The recovery of services during the second half of 2020 can be attributed to different reasons. In June 2020, the Federal Ministry of Health (FMoH) developed strategies to maintain essential health services and dissimilated guidance on service adaptation during COVID to the regions [36, 37]. In addition, since the number of COVID-19 cases and deaths were lower than initial expectations, public trust in receiving safe services from health facilities was improved. Hence, health facility visits increased overtime [38]. A number of health facilities also resumed various health services that had been suspended. Moreover, public hospitals introduced telemedicine in mid-2020. In collaboration with FMoH, health promotion campaigns were carried out by non-governmental organizations like UNICEF to maintain maternal and child health service use during the pandemic [39].

Nonetheless, the pandemic affected various services and these effects were largest in April-June 2020.This implies that the Ethiopian health system has a limited capacity to face shocks and was not prepared to maintain essential health services during a crisis [4]. The pandemic affected utilization of essential services directly and indirectly in various ways. First, COVID patients were given priority by central and local governments since coronavirus was considered to be a highly contagious disease which could lead to a high death rate. In response, there were closure of health facilities, reduction in services, and many health workers were redeployed to COVID-19 treatment centers [10, 40]. Second, there was fear of exposure to coronavirus while receiving care and many decided to forgo or delay care even when needed [41]. Third, as part of the state of emergency measures, the government also restricted movements of people for five months. This could have affected household income and financial capacity to afford healthcare. In addition, access to transport to travel to health facilities were also limited. In addition, several public hospitals in urban areas were converted in COVID treatment centers and stopped providing other health services. Other hospitals also reduced their level of inpatient admissions and cut the length of hospital stays to prevent COVID infections. This could have also reduced access to essential services in urban areas compared to pastoralist areas. On the contrary, the impact of COVID on utilization of services could be more persistent in pastoralist regions where access to medical services is extremely limited [42] Therefore, once COVID spreads in pastoralist areas, it would be difficult to provide treatment for both COVID patients and to those who need other types of services.

Similar to this evidence, a multi-country study based on data collected in Burkina Faso, Ethiopia, and Nigeria reported maternal health services were interrupted during the pandemic [4]. However, the percentage of reductions for these services were slightly less as compared to the reduction of other services including HIV treatments and surgical services. An assessment conducted in 11 Sub-Saharan African countries also indicated that the COVID-19 pandemic impaired utilization of services for 18% of the study respondents mainly due to travel restrictions, health facility closures, and fear of contracting the virus [43]. Another study stated that reduction in maternal health service use were less generalized even if significant declines in facility deliveries, antenatal care and postnatal care were observed in some Sub-Saharan African countries. In April 2020, there were about 18% reduction in antenatal care visit in Nigeria and a 17% decline in postnatal care use in Mali [2]. A study conducted in urban Ethiopia found a significant reduction in child vaccination services in urban area especially in Addis Ababa and Harari [30]. However, these reductions occurred only for a few months after the pandemic was declared.

Our study showed the effect of the COVID-19 pandemic on utilization of healthcare across Ethiopian regions using data from the DHIS2 for a period of two years and robust statistical methods. However, our study has the following limitations. First, the Ethiopian health information system continues to face challenges with data completeness, consistency and timeliness. Data quality may also vary across regions. For example, some have found higher odds of misreporting in Somali and Afar [44, 45]. Moreover, there is a risk that the pandemic affected the ability of facilities to report service utilization especially in those regions most heavily affected by the pandemic. We attempted to reduce estimation bias attributed to data quality problems by conducting two-step cleaning procedures in order to include only facilities with stable reporting over time and exclude outliers. This could affect generalizability by excluding the hardest hit facilities. However, we ensured that the data cleaning excluded less than 5% of service volumes provided over two years on average. Second, due to the conflict in Tigray, the results are not generalizable to that region. Third, the routine data systems generally did not include telemedicine consultations that were introduced to provide follow up services during the pandemic. Finally, due to aggregate reporting of service visits at regional levels, it is not possible to quantify the extent to which people switched from the public to the private sector during the pandemic.

## Conclusion

Within a month of the first confirmed COVID case, nearly all Sub-Saharan countries enforced swift and strict lock down measures at both national and regional levels. Such measures have contributed to contain the spread of COVID across the region [46, 47]. However, these restrictions brought unintended consequences by reducing the utilization of essential services [2, 3]. The evidence from our sub-national analysis in Ethiopia implies that health shocks especially affected those regions where there was community transmission. Reduction in health care utilization in Ethiopia could have prolonged and devastating effects. Therefore, it is necessary to design appropriate policies and strategies to strengthen the capacity of health systems to maintain essential health services during future pandemics or health shocks.

## Supporting information

S1 Appendix Table A. Sample size and confirmed COVID-19 cases per region. Table B. Effect of COVID on maternal and child health services across regions (ITSA—Regression with Newey-West standard errors). Table C. Effect of COVID on various services across regions (ITSA—Regression with Newey-West standard errors). Table D. List of healthcare use indicators included in the analysis and their definitions.
(DOCX)

S1 Fig Utilization of other health services in 2020 compared to 2019 by region type. (TIF)
