# Supplementary material for: Effect of the COVID-19 pandemic on health service utilization across regions of Ethiopia: An interrupted time series analysis of health information system data from 2019–2020
Source: PLOS Glob Public Health. 2022 Sep 12;2(9):e0000843. doi: 10.1371/journal.pgph.0000843 (PMC10021875; doi:10.1371/journal.pgph.0000843)
Supplement: S1 Appendix — Table A. Sample size and confirmed COVID-19 cases per region. Table B. Effect of COVID on maternal and child health services across regions (ITSA—Regression with Newey-West standard errors). Table C. Effect of COVID on various services across regions (ITSA—Regression with Newey-West standard errors). Table D. List of healthcare use indicators included in the analysis and their definitions. (DOCX) [file pgph.0000843.s001.docx]

**S1 Appendix**

**Table A. Sample size and confirmed COVID-19 cases per region**

| Region | Number of units reporting* | | COVID-19 cases in 2020** | | |  |
| --- | --- | --- | --- | --- | --- | --- |
|  | N | % | | N | % | |
| Addis Ababa | 315 | 16% | | 68,306 | 56% | |
| Afar | 46 | 2% | | 1,841 | 1% | |
| Amhara | 338 | 17% | | 6,573 | 5% | |
| Ben Gum | 24 | 1% | | 2,528 | 2% | |
| Dire Dawa | 19 | 1% | | 2,910 | 2% | |
| Gambella | 20 | 1% | | 1,008 | 1% | |
| Harari | 19 | 1% | | 2,814 | 2% | |
| Oromia | 646 | 33% | | 20,454 | 17% | |
| SNNP | 408 | 21% | | 8,093 | 7% | |
| Somali | 109 | 6% | | 1,675 | 1% | |
| National | 1944 | 100% | | 122,864 | 100% | |

* Units are either health facilities, Woreda Health Offices or Primary Health Center Units. Facility-level information was available for tertiary and secondary hospitals and private facilities and for all facilities in the capital, Addis Ababa. Data from other public facilities (primary hospitals, health centers and health posts) were aggregated and reported by Primary Health Center Units (in Dire Dawa and Harari regions) or by woreda health offices (in all other regions except Addis Ababa). The number of units reporting also varied by indicator.

**Confirmed Covid -19 cases are as of December 27th, 2020. The reported national figure includes cases confirmed in Tigray region which is coved in the study. Covid figures are from the National Public Health Emergency Operation Center (PHEOC) COVID-19 Weekly bulletin No.35.

**Table B. Effect of the COVID-19 pandemic on maternal and child health services across regions (ITSA - Regression with Newey-West standard errors)**

| **Region** | **STI visits** | | | **Postnatal care** | | | **BCG** | | | **OPV3** | | | |
| --- | --- | --- | --- | --- | --- | --- | --- | --- | --- | --- | --- | --- | --- |
|  | **Level change (95% CI)** | **Relative level change** | **Slope change (95% CI)** | **Level change (95% CI)** | **Relative level change** | **Slope change (95% CI)** | **Level change (95% CI)** | **Relative level change** | **Slope change (95% CI)** | **Level change (95% CI)** | **Relative level change** | **Slope change (95% CI)** |  |
| Addis Ababa | -1,234** (-1,671, -796) | -56% | 114** (48, 181) | -2,000** (-3,354, -646) | -19% | 45 (-285, 375) | -2,606 (-6,356, 1,145) | -23% | 574 (-52,1,199) | -1897** (-3,596, -199) | -18% | 103 (-143, 348) |  |
| Afar | -5 (-45, 36) | -4% | 3 (-3, 9) | -179 (-433, 76) | -8% | -6 (-36, 24) | -264 (-592, 63) | -8% | 22 (-33, 78) | -449** (-761, -138) | -14% | -4 (-54, 46) |  |
| Amhara | -1,616** (-2,259, -973) | -43% | 160** (52, -268) | -2,828 (-6,437, 781) | -7% | -63 (-573, 447) | 2,292 (-9,770, 14,355) | 4% | 128 (-1,400, 1,655) | -1,329 (-7,192, 4,534) | -3% | 134 (-809, 1,076) |  |
| Ben Gum | -32 (-69, 6) | -29% | -6 (-13, 1) | 310 (-170, 789) | 15% | -61 (-128, 7) | 118 (-598, 833) | 4% | -69 (-170, 32) | 128 (-297, 553) | 5% | -64 (-147, 20) |  |
| Dire Dawa | -7 (-60, 46) | -9% | 6 (-1, 13) | 40 (-106, 187) | 5% | 48** (27, 70) | -51 (-215, 112) | -5% | 45** (23, 68) | -309** (-416, -202) | -31% | 37** (21, 53) |  |
| Gambella | 41 (-52, 134) | 25% | 1 (-15, 18) | -6 (-102, 89) | -1% | -3 (-17, 11) | -30 (-128, 68) | -4% | 31** (17, 45) | -90 (-227, 47) | -9% | 36** (12, 60) |  |
| Harari | -17** (-31, -3) | -55% | 5** (2, 7) | -88 (-274, 97) | -13% | 75** (40, 110) | -156** (-305, -7) | -20% | 37** (19, 55) | -75(-195, 45) | -11% | 22 (-4, 49) |  |
| Oromia | -2,358** (-3,135, -1,581) | -32% | 292** (151, 432) | -4,996** (-9,174, -818) | -6% | 1,822** (1,304, 2,339) | -2,529 (-16,058, 11,000) | -2% | 2,773** (983, 4,563) | -8,231 (-17,413, 951) | -8% | 1,800** (466, 3,134) |  |
| SNNP | -791** (-1,094, -487) | -38% | 85** (49, 121) | -1,159 (-3,004, 687) | -2% | -9 (-252, 235) | 4,081 (-2,495, 10,658) | 8% | 504 (-441, 1,448) | -1,079 (-5,162, 3,003) | -2% | 271 (-360, 901) |  |
| Somali | -35 (-151, 81) | -36% | -7 (-19, 4) | -577 (-1,333, 180) | -7% | -65 (-171, 41) | 91 (-709, 891) | 1% | -126** (-239, -13) | -522 (-1,607, 563) | -4% | -427** (-573, -281) |  |

Level change shows the immediate effect of the pandemic in April 2020 and slope change is the monthly change during COVID-19 until the end of 2020. Relative level change is the immediate level change in April 2020 compared to the average during the pre-COVID period (January 2019 to March 2020). Asterisks ** indicate statistical significance (p ≤0.05). SNNP is Southern Nations, Nationalities and People’s region. Ben-Gum is Benishangul-Gumuz region.

**Table C. Effect of the COVID-19 pandemic on various services across regions (ITSA - Regression with Newey-West standard errors)**

| **Region** | **Pneumococcal** | | | **Rotavirus** | | | **People on ART** | | | | **Road traffic accidents** | | | |
| --- | --- | --- | --- | --- | --- | --- | --- | --- | --- | --- | --- | --- | --- | --- |
|  | **Level change (95% CI)** | **Relative level change** | **Slope change (95% CI)** | **Level change (95% CI)** | **Relative level change** | **Slope change (95% CI)** | **Level change (95% CI)** | **Relative level change** | **Slope change (95% CI)** | **Level change (95% CI)** | | **Relative level change** | **Slope change (95% CI)** |  |
| Addis Ababa | -963 (-2,542, 616) | -9% | 38 (-216, 293) | 64 (-1,427, 1,554) | 1% | -45 (-295, 205) | 633 (-1,820, 3,085) | 1% | -212 (-703, 279) | -175 (-352, 2) | | -27% | 25 (-4, 54) |  |
| Afar | -319** (-589, -48) | -10% | 9 (-42, 59) | -426** (-724, -129) | -13% | 44** (7, 81) | 216 (-286, 718) | 4% | -8 (-95, 79) | -54** (-106, -2) | | -39% | -6 (-13, 1) |  |
| Amhara | -1,131 (-7,021, 4,759) | -2% | 130 (-819, 1,079) | -267 (-4,696, 4,162) | -1% | -11 (-699, 676) | -105 (-4,031, 3,821) | 0% | -26 (-461, 409) | -478** (-717, -238) | | -30% | 64** (25, 104) |  |
| Ben Gum | 130 (-294, 554) | 5% | -61 (-144, 23) | 253 (-175, 681) | 10% | -83** (-158, -7) | 76 (-45, 198) | 2% | -7 (-29, 16) | -17 (-40, 7) | | -39% | 3 (-2, 8) |  |
| Dire Dawa | -290** (-393, -187) | -29% | 39** (23, 55) | -262** (-336, -187) | -26% | 41** (32 ,51) | 32 (-362, 426) | 1% | -8 (-82, 65) | -52** (-105, -0) | | -37% | 8 (-1, 16) |  |
| Gambella | -44 (-160, 71) | -5% | 39** (21, 58) | -78 (-224, 69) | -8% | 35** (11, 59) | -1,009** (-1,899, -118) | -16% | 125 (-8, 258) | 16 (-8, 39) | | 129% | -1 (-4, 3) |  |
| Harari | -119 (-241, 3) | -18% | 28** (9, 47) | -142** (-247, -36) | -21% | 31** (17, 46) | 389 (-379, 1,156) | 9% | -27 (-101, 48) | -91** (-139, -42) | | -92% | 10** (1, 19) |  |
| Oromia | -6,785 (-15,198, 1,628) | -6% | 1,776** (532, 3,020) | -5,083 (-15,451, 5,284) | -5% | 1,354 (-228, 2,935) | -508 (-2,376, 1,361) | 0% | 497** (251, 742) | -988** (-1,643, -334) | | -18% | 123** (23, 223) |  |
| SNNP | 151 (-4,707, 5,009) | 0% | 158 (-612, 929) | 654 (-4,120, 5,428) | 1% | 168 (-584, 919) | -846 (-2,590, 898) | -2% | 334** (75, 593) | -807 (-2,282, 668) | | -9% | -50 (-226, 127) |  |
| Somali | -671 (-1,734, 392) | -5% | -446** (-593, -299) | -841 (-1,694, 12) | -6% | -437** (-563, -312) | -599 (-1,368, 171) | -33% | 85 (-39, 209) | 68 (-6, 143) | | 24% | -11 (-27, 5) |  |

Level change shows the immediate effect of the pandemic in April 2020 and slope change is the monthly change during COVID-19 until the end of 2020. Relative level change is the immediate level change in April 2020 compared to the average during the pre-COVID period (January 2019 to March 2020). Asterisks ** indicate statistical significance (p ≤0.05). SNNP is Southern Nations, Nationalities and People’s region. Ben-Gum is Benishangul-Gumuz region.

**Table D. List of healthcare use indicators included in the analysis and their definitions**

| **Type of service** | **indicator** | Definition |
| --- | --- | --- |
| **Summative measures** | **Outpatient visits** | Number of outpatient visits |
|  | **Emergency room visits** | Total number of emergency unit attendances |
|  | **Inpatient admissions** | Number of inpatient admissions |
| **Reproductive and maternal** | **Contraceptive users** | Number of new and current users of contraceptives |
|  | **STI visits** | Number of consultations for STI care |
|  | **Antenatal care** | Number of pregnant women that received ANC at least once by maternal age |
|  | **Deliveries** | Total number of births attended by skilled health personnel |
|  | **Caesarean sections** | Number of women having given birth by caesarean section |
| **Child health** | **Postnatal care** | Number of postnatal visits within 7 days of delivery |
|  | **Diarrhea** | Number of children treated for diarrhea with ORS only |
|  | **Malnutrition** | Total number of children <5yrs screened for acute malnutrition |
|  | **Pneumonia** | Number of children under 5 treated for pneumonia |
| **Child vaccinations** | **BCG** | Number of children under one year of age who have received BCG vaccine |
|  | **Pentavalent** | Number of children under one year who have received third dose of pentavalent vaccine |
|  | **OPV3** | Number of children under one year of age who have received third dose of polio vaccine |
|  | **Pneumococcal** | Number of children under one year of age who have received third dose of pneumococcal vaccine |
|  | **Rotavirus** | Number of children under one year of age who have received 2nd dose of Rotavirus vaccine |
|  | **Measles** | Number of children under one year who received first dose of measles vaccine |
|  | **Fully vaccinated by 1** | Number of children received all vaccine doses before 1st birthday |
| **Other services** | **People on ART** | Number of adults and children who are currently on ART |
|  | **Road traffic accidents** | Number of road traffic injury cases disaggregated by accident type |
