# Supplementary figures and images for: Effect of the COVID-19 pandemic on health service utilization across regions of Ethiopia: An interrupted time series analysis of health information system data from 2019–2020
Source: PLOS Glob Public Health. 2022 Sep 12;2(9):e0000843. doi: 10.1371/journal.pgph.0000843 (PMC10021875; doi:10.1371/journal.pgph.0000843)

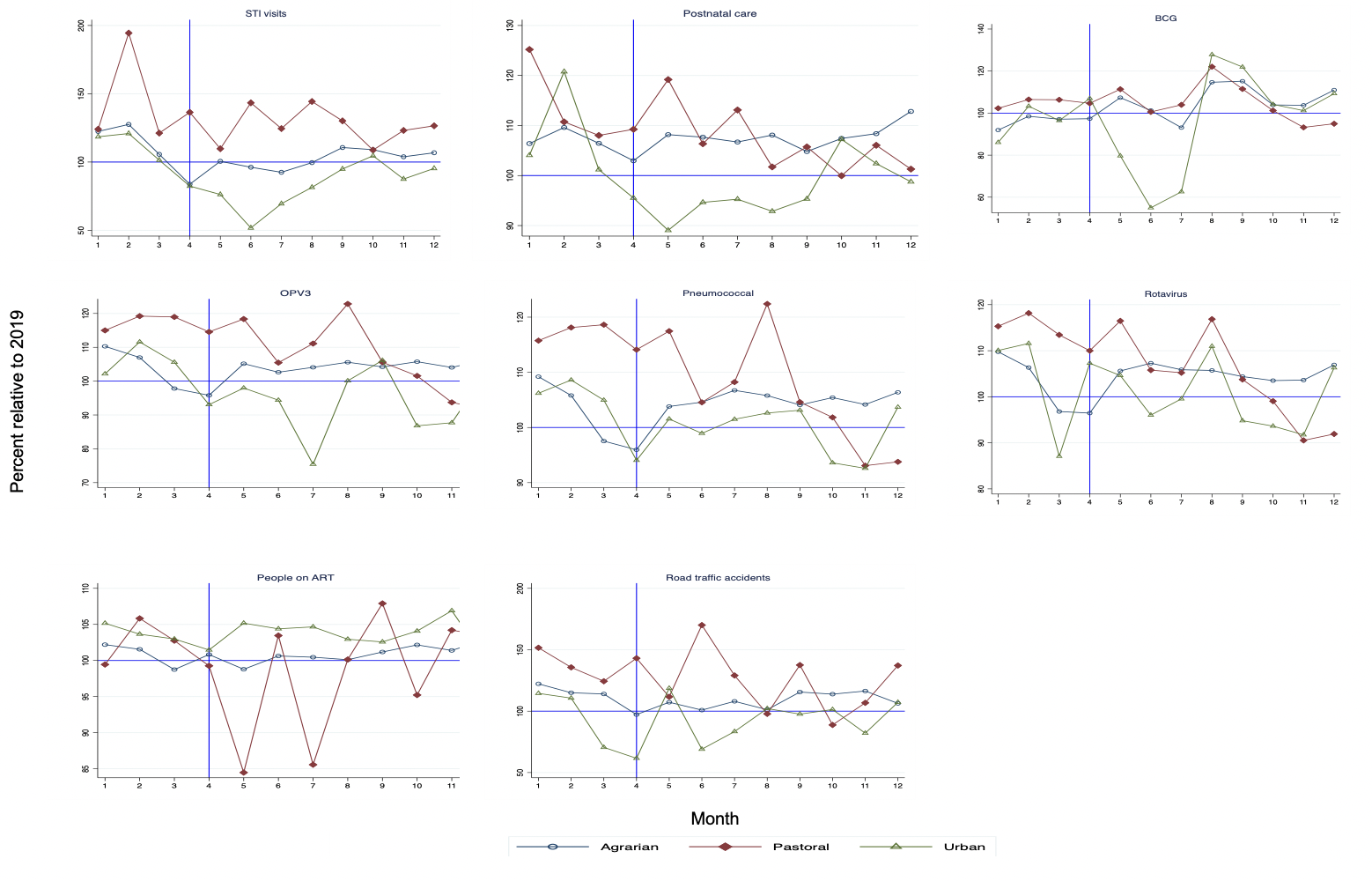

Supplement: S1 Fig — (TIF) [file pgph.0000843.s002.tif]
